# Evaluating and enhancing the service capacity of secondary public hospitals in urban China: a multi-method empirical analysis based on Guangzhou (2019–2023)

**DOI:** 10.3389/frhs.2025.1621018

**Published:** 2025-06-12

**Authors:** Baoling Wu, Wenbo Wu, Xi Wang, Weizhang Huang, Zhenni Luo, Jifeng Li

**Affiliations:** ^1^ The Second Affiliated Hospital, Guangzhou Medical University, Guangzhou, Guangdong, China; ^2^ Faculty of Humanities and Social Sciences, Macau Polytechnic University, Macau, China; ^3^ School of Health Management, Guangzhou Medical University, Guangzhou, Guangdong, China; ^4^ School of Public Health, Guangzhou Medical University, Guangzhou, Guangdong, China

**Keywords:** secondary public hospitals, healthcare service capacity evaluation, entropy-topsis, kernel density estimation, Dagum Gini coefficient

## Abstract

**Background:**

Secondary public hospitals play a pivotal role in China's hierarchical medical system, serving as a critical intermediary tier. However, in rapidly urbanizing cities such as Guangzhou, these hospitals face mounting challenges including widening efficiency disparities, imbalanced resource allocation, and weak governance structures. This study aims to systematically evaluate the evolution and spatial dynamics of service capacity among secondary general public hospitals in Guangzhou, offering empirical evidence to support capacity improvement and policy optimization.

**Methods:**

A composite evaluation framework was constructed across three dimensions: medical quality, operational efficiency, and sustainability. Based on panel data from 12 secondary general public hospitals in Guangzhou between 2019 and 2023, we applied a combination of Entropy-TOPSIS model, Kernel Density Estimation (KDE), and the Dagum Gini Coefficient to assess overall service capacity levels, temporal trends, and spatial inequalities.

**Results:**

The findings indicate a general upward trend in service capacity; however, disparities among hospitals have intensified. While indicators of medical safety (e.g., mortality and complication rates) have steadily improved, there remains significant divergence in surgical ratios and pharmaceutical service coverage—particularly in peripheral areas. KDE analysis reveals a transition from unimodal to bimodal distribution, indicating stratification of service capacity. Decomposition of the Dagum Gini Coefficient shows that transvariation (inter-group overlaps) is the main source of inequality, underscoring increasing cross-regional capacity divergence.

**Conclusions:**

Although Guangzhou's secondary public hospitals have shown overall improvement, challenges remain in terms of regional coordination and internal structural disparities. This study recommends differentiated interventions such as specialty alliances, performance-based resource allocation, and workforce optimization to enhance system resilience and equity. The proposed evaluation model demonstrates strong applicability and scalability, offering theoretical and empirical insights for healthcare system governance in other rapidly urbanizing regions.

## 1 Introduction

Amid the deepening reform of global health systems, enhancing the service capacity of public hospitals—particularly secondary-level general public hospitals in urban areas—has become a focal issue in health policy development worldwide. These hospitals, positioned between primary care institutions and tertiary hospitals, are essential for receiving referrals from the community level and managing moderately complex conditions. They serve a bridging role within China's hierarchical diagnosis and treatment system.

However, in the context of rapid urbanization, secondary hospitals in many developing countries face significant challenges, including underdeveloped resource allocation and insufficient attention to their service capacity. Studies have shown that in Nanjing, China, for example, healthcare resources are unevenly distributed, with high concentration in central districts and much weaker capacity in peripheral areas (1). In Nigeria, urbanization has resulted in medical personnel migrating toward cities, thereby reducing healthcare accessibility in rural areas—especially for the elderly (2). Similarly, in Thailand, urban expansion has widened the accessibility gap between urban and rural medical facilities, highlighting the need for spatial planning interventions to ensure more equitable resource distribution (3). These cases reflect a common challenge: urbanization places significant pressure on the equity and sustainability of healthcare systems.

To respond to these challenges, the global community is increasingly recognizing the need to strengthen hospital service capacity, especially by empowering “middle-tier” institutions. As urbanization alters the structure of healthcare demand, scientifically evaluating and optimizing resource allocation and hospital capacity has become a shared concern among health policymakers.

In recent years, various international organizations and research institutes have introduced frameworks to support performance-based resource planning. The World Health Organization (WHO) has promoted performance-oriented allocation models that emphasize service integration to improve the efficiency of healthcare systems. Strengthening secondary hospitals is a key part of this effort, with WHO advocating for them to act as regional service “hubs” that support integration with primary care and public health services, thereby optimizing resource use for specific populations (4). In 2003, the WHO Regional Office for Europe developed the Performance Assessment Tool for Quality Improvement in Hospitals (PATH), which evaluates hospital performance across dimensions such as clinical effectiveness, efficiency, and staff orientation. After being piloted in six countries between 2004 and 2006, PATH expanded in 2007–2008 to 140 hospitals across eight European countries including Belgium, France, and Germany (5, 6). In 2010, the WHO and USAID jointly launched the Service Availability and Readiness Assessment (SARA), offering manuals (7) and implementation guides (8) to assess the accessibility and readiness of health services. This framework has since been adopted in Bangladesh, Burkina Faso, Ghana, Mozambique, and other countries (9–13).

Meanwhile, methodological innovations in healthcare capacity evaluation are trending toward diversification and integration. These methods are widely used in assessing service efficiency, optimizing resource allocation, and analyzing regional equity. Multi-Criteria Decision Analysis (MCDA) approaches, including entropy-weighted TOPSIS, Analytic Hierarchy Process (AHP), and Stochastic Frontier Analysis (SFA), have been broadly applied in hospital performance evaluation and healthcare efficiency comparisons (14, 15).

In pursuit of Sustainable Development Goal 3—“Ensure healthy lives and promote well-being for all at all ages”—scholars are increasingly integrating spatial and inequality analytics. For example, Kernel Density Estimation (KDE), a non-parametric method, has been widely used to explore spatial clustering and evolution of healthcare capacity (16). In contrast, the Dagum Gini coefficient offers decomposable metrics that allow more detailed examination of within-region, between-region, and transvariation (overlap) inequality, outperforming the traditional Gini index in explaining structural disparities (17).

China's healthcare system is structured into three levels, with secondary general public hospitals playing a central role in the implementation of hierarchical diagnosis and treatment. These hospitals primarily serve urban and peri-urban populations by offering general diagnostic, inpatient, and outpatient care. They are also essential in public health initiatives such as chronic disease management and epidemic response. However, compared with tertiary hospitals, secondary hospitals often fall into a “resource valley”—marked by shortages in manpower, equipment, and policy attention. Moreover, their contribution is frequently overlooked in policy prioritization and funding, which leads to weakened functional roles. Although academic interest in secondary hospitals has grown, most studies remain focused on single-year financial or performance metrics, lacking insight into the dynamic evolution of capacity or structural inequality—especially in megacities with pronounced development imbalances (18, 19). Furthermore, the COVID-19 pandemic exposed the vulnerabilities of hospital systems, prompting a re-evaluation of the resilience and load-bearing capacity of non-core hospitals. Secondary hospitals played a crucial role in initial diagnosis and triage, yet lacked the tertiary hospitals’ infrastructure and resources—highlighting the need for more refined evaluation tools and targeted governance strategies (20, 21).

In light of this context, this study focuses on Guangzhou, a major city in South China, and examines 12 secondary general public hospitals from 2019 to 2023. A comprehensive evaluation framework is developed based on three key dimensions:

• Medical Quality (e.g., average length of stay, surgical complication rates),
• Operational Efficiency (e.g., income structure, cost containment),
• Sustainability (e.g., staffing ratios, physician workload intensity).

Methodologically, the study integrates three analytical tools:

• Entropy-weighted TOPSIS, which enables objective weighting and ranking of hospitals based on service capacity;
• Kernel Density Estimation (KDE), to examine the evolution of score distributions over time;
• Dagum Gini Coefficient decomposition, to measure and decompose spatial inequality in service capacity.

By triangulating results from these methodologies, this study proposes a replicable and transferable evaluation framework tailored to secondary hospitals in urban China. It aims to support local policy evaluation in Guangzhou and offer insights for improving healthcare system governance in other rapidly urbanizing regions. In addressing the global challenges of “filling the gaps” and “strengthening mid-tier institutions,” this study seeks to contribute reform strategies and analytical methods that are both empirically grounded and globally applicable.

## 2 Materials and methods

### 2.1 Data sources

This study targets 12 secondary general public hospitals located within the administrative boundaries of Guangzhou. A comprehensive evaluation index system was developed to assess their healthcare service capacity (see Table 1). The index system is structured into three primary dimensions, each representing a core pillar of institutional performance:

• Medical Quality: This dimension evaluates clinical outcomes and safety. Indicators include average length of stay, low-risk group mortality rate, surgical rate, complication rate. These measures reflect the hospital's ability to deliver safe, effective, and timely care—essential to meeting patient expectations and improving health outcomes.
• Operational Efficiency: This component assesses resource use and cost containment. It includes the ratio of service revenue to total revenue, personnel cost ratio, asset-liability ratio, outpatient and inpatient cost growth. These indicators are crucial for evaluating financial sustainability and responsiveness to health payment reforms, such as Diagnosis-Related Group (DRG)–based funding.
• Sustainability: This dimension captures human resource adequacy and service resilience. It includes nurse-to-doctor ratio, physician workload, pharmacist staffing. These indicators help identify whether hospitals possess the staffing capacity to maintain stable service delivery under both routine and emergency conditions.

**Table 1:** Evaluation index system for healthcare service capacity of secondary public hospitals.

Primary dimension	Secondary indicator	Unit	Indicator orientation	Code
Medical quality	Average length of stay	Days	Negative	$X_{1}$
	Mortality rate of low-risk patients	%	Negative	$X_{2}$
	Proportion of discharged patients who underwent surgery	%	Positive	$X_{3}$
	Complication rate among surgical patients	%	Negative	$X_{4}$
Operational efficiency	Proportion of medical service revenue (excluding drugs, consumables, and diagnostic fees)	%	Positive	$X_{5}$
	Personnel expenditure as a proportion of operational expenditure	%	Positive	$X_{6}$
	Asset-liability ratio	%	Negative	$X_{7}$
	Growth rate of per outpatient visit cost	%	Negative	$X_{8}$
	Growth rate of per outpatient drug cost	%	Negative	$X_{9}$
	Growth rate of per inpatient cost	%	Negative	$X_{10}$
	Growth rate of per inpatient drug cost	%	Negative	$X_{11}$
Sustainability	Nurse-to-doctor ratio	%	Positive	$X_{12}$
	Average daily inpatient workload per licensed physician	Bed-days	Negative	$X_{13}$
	Number of pharmacists per 100 hospital beds	Persons	Positive	$X_{14}$

The selection of these three dimensions ensures a balanced and policy-relevant assessment framework. It integrates clinical effectiveness, economic performance, and long-term institutional resilience—aligning with national healthcare reform goals and international standards in health systems evaluation.

Each secondary indicator is defined with its unit, orientation (positive/negative), and coded numerically ($X_{1}$–$X_{14}$). The indicators collectively form a multidimensional representation of hospital service capacity. Data were drawn from multiple sources spanning 2019 to 2023, including the *Guangzhou Statistical Yearbook*, official reports from the Guangzhou Health Commission, and municipal government publications.

### 2.2 Research methods

To ensure robustness and multidimensional insights, three quantitative methods were employed:

#### 2.2.1 Entropy weight-TOPSIS method

The Entropy-Weighted TOPSIS method integrates entropy-based weight assignment with the TOPSIS (Technique for Order Preference by Similarity to Ideal Solution) approach to handle multi-criteria decision-making problems (22–24). It addresses challenges associated with subjective weighting and correlation among indicators, and is widely applied in healthcare, construction, and industrial evaluation scenarios (23, 25–27, 28). Stepwise procedure:

Step 1: Normalization of the Original Data Matrix

Let there be *n* evaluation objects (e.g., hospitals) and *m* evaluation indicators. The original data matrix is denoted as:

$$X=\left\{\begin{array}{cccc} x_{11} & x_{12} & \ldots & x_{1m}\\ x_{21} & x_{22} & \ldots & x_{2m}\\ \ldots & \ldots & \ldots & \ldots \\ x_{n1} & x_{n2} & \ldots & x_{nm} \end{array}\right\}n\times m$$

To standardize the original matrix *X*, we apply min-max normalization to obtain the normalized matrix $R=(r_{ij})n\times m$, where $r_{ij}\in [0,1]$ represents the normalized score of the *i* -th object on the *j*-th indicator:

• For positive indicators (the higher, the better): $r_{ij}=\frac{x_{ij}-\min(x_{j})}{\max(x_{j})-\min(x_{j})}$
• For negative indicators (the lower, the better): $r_{ij}=\frac{\max(x_{j})-x_{ij}}{\max(x_{j})-\min(x_{j})}$
• Step 2: Entropy-Based Weight Calculation and Weighted Matrix Construction

Let $l_{ij}$ ​ denote the normalized proportion of indicator *j* for object *i*, computed from matrix R:

$$l_{ij}=\frac{r_{ij}}{\sum_{i=1}^{n}r_{ij}}$$

Define $e_{j}$ ​ as the entropy value for indicator *j*. The weight $w_{j}$ ​ is then calculated as:

$w_{j}=\frac{1-e_{j}}{\sum_{\;j=1}^{m}(1-e_{j})}$, $0\leq w_{j}\leq 1$, $\overset{m}{\underset{\;j=1}{\sum }}w_{j}=1$

The weighted normalized matrix *Z* is obtained by:

$$Z=w_{j}\times R=\left\{\begin{array}{cccc} w_{1}r_{11} & w_{2}r_{12} & \ldots & w_{m}r_{1m}\\ w_{1}r_{21} & w_{2}r_{22} & \ldots & w_{m}r_{2m}\\ \ldots & \ldots & \ldots & \ldots \\ w_{1}r_{n1} & w_{2}r_{n2} & \ldots & w_{m}r_{nm} \end{array}\right\}=\left\{\begin{array}{cccc} z_{11} & z_{12} & \ldots & z_{1m}\\ z_{21} & z_{22} & \ldots & z_{2m}\\ \ldots & \ldots & \ldots & \ldots \\ z_{n1} & z_{n2} & \ldots & z_{nm} \end{array}\right\}$$

Step 3: Identify Ideal and Anti-Ideal Solutions

For each indicator *j*, determine the best (ideal) and worst (anti-ideal) values:

• Ideal solution: $z_{j}^{+}=\max(z_{\;jj})$, thus $z_{j}^{+}=\;(z_{1}^{+},z_{2}^{+},\ldots ,z_{m}^{+})$
• Anti-ideal solution: $z_{j}^{-}=\min(z_{\;jj})$, thus $z_{j}^{-}=\;(z_{1}^{-},z_{2}^{-},\ldots ,z_{m}^{-})$
• Step 4: Calculate Euclidean Distances

For each evaluation object *i*, compute its distance to the ideal and anti-ideal solutions:

• Distance to ideal: $d_{i}^{+}=\sqrt{\overset{m}{\underset{\;j=1}{\sum }}{\left(z_{ij}-z_{j}^{+}\right)}^{2}}$
• Distance to anti-ideal: $d_{i}^{-}=\sqrt{\overset{m}{\underset{\;j=1}{\sum }}{\left(z_{ij}-z_{j}^{-}\right)}^{2}}$
• Step 5: Compute Relative Closeness to the Ideal Solution

The relative closeness coefficient $v_{i}$ ​ is calculated as:

$$v_{i}=\frac{d_{i}^{-}}{d_{i}^{+}+d_{i}^{-}},\;0\leq v_{i}\leq 1$$

A higher $v_{i}$ ​ indicates stronger service capacity of the corresponding hospital. The ranking of $v_{i}$ ​ values reflects the comparative performance across all evaluated hospitals.

#### 2.2.2 Kernel density estimation (KDE)

KDE is a non-parametric method for estimating the probability density function of a continuous variable, offering flexibility in modeling data distributions without assuming a parametric form (29–31). It is especially valuable in visualizing temporal evolution and regional heterogeneity in healthcare quality assessments (32–34).

The Gaussian kernel function is adopted:

$$\hat{\;f_{h}}(x)=\frac{1}{\mathrm{nh}}\overset{n}{\underset{n=1}{\sum }}K\left(\frac{x_{i}-\bar{x}}{h}\right)$$

where K(⋅) is the Gaussian kernel, *h* is the bandwidth, and *n* is the sample size.

KDE allows the visualization of capacity distribution shifts and stratification patterns across years, highlighting convergence or divergence trends.

#### 2.2.3 Dagum Gini coefficient decomposition

The Dagum Gini coefficient, proposed by Argentine-Canadian economist Camilo Dagum in the 1970s, represents a significant advancement over the traditional Gini index. It is based on the three-parameter Dagum probability distribution, which offers superior fitting performance—particularly in capturing inequalities at both tails of a distribution. Its key advantage lies in its decomposability, enabling the total inequality to be separated into within-group inequality, between-group inequality, and transvariation (overlapping) inequality components. This makes the Dagum Gini model a powerful analytical tool for examining the structural dimensions of inequality (35, 36). Due to its enhanced explanatory capacity, it has been widely applied across economics, social sciences, public policy, and healthcare resource allocation to investigate the deep structures and dynamic mechanisms underlying inequality (37, 38).

In this study, the Dagum Gini coefficient is applied using subgroup decomposition to assess the spatial differentiation in healthcare service capacity among secondary public general hospitals in Guangzhou. The total inequality *G* is composed of the following three components:

$$G=G_{w}+G_{nb}+G_{t}$$

The respective formulas are defined as follows:

• Total Gini Coefficient:
  $$G=\frac{\sum_{\;j=1}^{k}\sum_{h=1}^{k}\sum_{i=1}^{n_{j}}\sum_{r=1}^{n_{h}}|y_{\;ji}-y_{hr}|}{2n^{2}\bar{y}}$$
• Within-Region Inequality:
  $$G_{\;jj}=\frac{\sum_{i=1}^{n_{j}}\sum_{r=1}^{nj}|y_{\;ji}-y_{hr}|}{2\bar{y_{j}}n_{j}^{2}}$$
• Between-Region Inequality:
  $$G_{\;jh}=\frac{\sum_{i=1}^{n_{j}}\sum_{r=1}^{nh}|y_{\;ji}-y_{hr}|}{n_{j}n_{h}(\bar{y_{j}}+\bar{y_{h}})}$$
• Decomposition of Within-Region Inequality:
  $$G_{w}=\overset{k}{\underset{\;j=1}{\sum }}G_{\;jj}p_{j}s_{j}$$
• Decomposition of Between-Region Inequality:
  $$G_{nb}=\overset{k}{\underset{\;j=2}{\sum }}\overset{\;j-1}{\underset{h=1}{\sum }}G_{\;jh}(\;p_{j}s_{h}+p_{h}s_{j})D_{\;jh}$$
• Transvariation (Overlapping Component):
  $$G_{t}=\overset{k}{\underset{\;j=2}{\sum }}\overset{\;j-1}{\underset{h=1}{\sum }}G_{\;jh}(\;p_{j}s_{h}+p_{h}s_{j})(1-D_{\;jh})$$

Where $y_{\;ji}$ ​ and $y_{hr}$ ​ denote the healthcare service capacity of hospital *i* in region *j* and hospital *r* in region *h*, respectively. $\bar{y}$ ​ represents the overall average service capacity across all hospitals, while ${\bar{y}}_{j}$ denotes the mean capacity within region *j*. *n* is the total number of hospitals, and $n_{j}$ ​, $n_{h}$ ​ are the number of hospitals in regions *j* and *h*, respectively. The term $p_{j}=\frac{n_{j}}{n}$ indicates the proportion of hospitals in region *j*, and $s_{j}=\frac{n_{\;j\;}\bar{y_{j}}}{n\bar{y}}$ reflects the weighted contribution of region *j* to the overall capacity level. $D_{\;jh}$ ​ is a directional influence index that measures the relative dominance of region *j* compared to region *h* in terms of healthcare service capacity.

## 3 Results and analysis

### 3.1 Descriptive analysis of Key indicators

Based on the statistical summary in Table 2, the healthcare service capacity of Guangzhou's 12 secondary general public hospitals demonstrated a combination of progress and divergence across the three key dimensions between 2019 and 2023.

**Table 2:** Descriptive statistics of Key evaluation indicators for 12 secondary public hospitals in Guangzhou (2019–2023).

Code	Indicator	2019	2020	2021	2022	2023
$X_{1}$	Average length of stay (days)	7.93 ± 2.56	9.18 ± 2.45	9.18 ± 2.55	8.94 ± 2.54	9.68 ± 2.78
$X_{2}$	Mortality rate of low-risk patients (%)	1.00 ± 0.94	0.59 ± 0.37	0.42 ± 0.33	0.29 ± 0.15	0.25 ± 0.11
$X_{3}$	Surgical discharge ratio (%)	21.66 ± 9.16	26.97 ± 9.30	24.57 ± 9.80	23.02 ± 9.01	16.88 ± 5.78
$X_{4}$	Complication rate among surgical patients (%)	0.64 ± 0.31	0.57 ± 0.32	0.50 ± 0.29	0.51 ± 0.32	0.46 ± 0.29
$X_{5}$	Proportion of service revenue (non-drug, non-consumable, %)	33.88 ± 4.76	34.21 ± 3.75	33.66 ± 4.24	33.54 ± 3.38	33.78 ± 4.04
$X_{6}$	Personnel cost ratio (%)	50.42 ± 4.13	47.11 ± 4.30	45.61 ± 5.21	43.09 ± 5.28	39.53 ± 6.24
$X_{7}$	Asset-liability ratio (%)	28.62 ± 6.79	29.18 ± 6.33	28.84 ± 6.49	28.95 ± 6.64	27.49 ± 7.31
$X_{8}$	Outpatient cost growth rate (%)	2.73 ± 4.52	3.37 ± 3.70	4.79 ± 3.98	5.25 ± 4.12	41.77 ± 20.86
$X_{9}$	Outpatient drug cost growth rate (%)	3.06 ± 5.81	2.54 ± 5.17	4.66 ± 4.77	4.15 ± 4.69	43.94 ± 21.57
$X_{10}$	Inpatient cost growth rate (%)	6.58 ± 5.31	7.35 ± 4.74	−2.69 ± 6.17	−2.18 ± 5.87	−3.17 ± 6.99
$X_{11}$	Inpatient drug cost growth rate (%)	6.17 ± 6.09	7.01 ± 5.33	−1.83 ± 5.64	−2.10 ± 5.13	−2.73 ± 6.44
$X_{12}$	Nurse-to-doctor ratio (%)	1.44 ± 0.24	1.50 ± 0.26	1.52 ± 0.24	1.54 ± 0.25	1.59 ± 0.25
$X_{13}$	Inpatient workload per physician (bed-days/day)	3.23 ± 0.51	3.22 ± 0.49	3.18 ± 0.50	3.13 ± 0.49	3.11 ± 0.47
$X_{14}$	Pharmacists per 100 beds (persons)	4.29 ± 1.20	4.17 ± 1.08	4.01 ± 0.96	3.98 ± 0.91	3.95 ± 0.93

Values are presented as mean ± standard deviation.

#### 3.1.1 Medical quality (*X*_1_–*X*_4_)

• The average length of stay ($X_{1}$) increased from 7.93 days in 2019 to 9.68 days in 2023, indicating a slowdown in patient turnover efficiency. The substantial rise during 2020–2021 corresponds to COVID-19 control policies that extended hospitalization duration. The increasing standard deviation over time suggests growing variability in treatment efficiency among hospitals.
• The mortality rate for low-risk patients ($X_{2}$) dropped sharply from 0.999% to 0.247%, alongside a dramatic decrease in variance. This reflects notable improvements in medical safety and standardization in treatment practices across facilities.
• The surgical discharge ratio ($X_{3}$), which peaked at 26.97% in 2020, dropped significantly to 16.88% in 2023. This could be attributed to shifts in disease case-mix, surgical policy adjustments, or redistribution of surgical resources during the pandemic.
• The surgical complication rate ($X_{4}$) decreased modestly throughout the period, indicating an overall improvement in surgical safety and intraoperative risk management.

#### 3.1.2 Operational efficiency (*X*_5_–*X*_11_)

• The proportion of service revenue ($X_{5}$) remained relatively stable (∼33.5%), reflecting consistent reliance on core medical services over drugs or diagnostics.
• The personnel cost ratio ($X_{6}$) declined steadily from 50.42% to 39.53%, suggesting improved financial efficiency. However, increasing standard deviation indicates widening disparity in cost control strategies across hospitals.
• The asset-liability ratio ($X_{7}$) remained stable with a slight downward trend in 2023, but high dispersion points to unequal debt structures and financial pressure between institutions.
• The growth rates of outpatient costs ($X_{8}$) and outpatient drug costs ($X_{9}$) surged significantly in 2023, exceeding 40%. Such spikes imply possible lapses in cost regulation or changes in insurance pricing structures.
• Meanwhile, inpatient cost ($X_{10}$) and drug cost growth ($X_{11}$) both entered negative territory post-2021. This aligns with national efforts to reduce hospitalization expenditures under DRG and insurance payment reforms.

#### 3.1.3 Sustainability (*X*_12_–*X*_14_)

• The nurse-to-doctor ratio ($X_{12}$) improved incrementally, reflecting minor adjustments in workforce composition and possibly enhanced nursing investment.
• The average daily inpatient workload per physician ($X_{13}$) showed a steady decline, suggesting a reduction in individual burden, although at the potential cost of system efficiency.
• The pharmacist-to-bed ratio ($X_{14}$) continued its downward trend, raising concerns over pharmaceutical care capacity and drug-use governance.

### 3.2 Comprehensive evaluation via entropy-TOPSIS

#### 3.2.1 Indicator weights and discrimination power

As presented in Table 3, the entropy analysis yielded consistently high values for most indicators $\left(e_{j}>0.95\right)$, suggesting strong uniformity of hospital performance in many dimensions. However, certain variables exhibited greater discriminative potential. Surgical discharge ratio ($X_{3}$) showed a relatively low entropy and the highest weight (17.46%), indicating its substantial role in differentiating service complexity and procedural capabilities among hospitals. Pharmacists per 100 beds ($X_{14}$) ranked second in weight (16.03%), reflecting the importance of pharmaceutical staffing in assessing service comprehensiveness and support quality. Inpatient drug cost growth rate ($X_{11}$) held the third highest weight (13.06%), highlighting the growing relevance of cost control and insurance adaptability as policy pressure on inpatient costs intensifies. Conversely, outpatient drug cost growth ($X_{9}$) and surgical complication rate ($X_{4}$), despite high entropy values, received minimal weight due to excessive variance or weak inter-hospital differentiation, suggesting limited evaluation utility under current measurement conditions.

**Table 3:** Entropy values and weights of service capacity evaluation indicators (2019–2023).

Code	Indicator	Entropy value ($e_{j}$)	Entropy weight ($w_{j}$)
$X_{1}$	Average length of stay (days)	0.9574	0.0278
$X_{2}$	Mortality rate of low-risk patients (%)	0.9689	0.0198
$X_{3}$	Surgical discharge ratio (%)	0.9127	0.1746
$X_{4}$	Complication rate among surgical patients (%)	0.9785	0.0142
$X_{5}$	Proportion of service revenue (non-drug, non-consumable, %)	0.9564	0.0287
$X_{6}$	Personnel cost ratio (%)	0.9511	0.0321
$X_{7}$	Asset-liability ratio (%)	0.9623	0.0236
$X_{8}$	Outpatient cost growth rate (%)	0.9596	0.0253
$X_{9}$	Outpatient drug cost growth rate (%)	0.9796	0.0134
$X_{10}$	Inpatient cost growth rate (%)	0.9563	0.0288
$X_{11}$	Inpatient drug cost growth rate (%)	0.9337	0.1306
$X_{12}$	Nurse-to-doctor ratio (%)	0.9495	0.0335
$X_{13}$	Inpatient workload per physician (bed-days/day)	0.9649	0.0213
$X_{14}$	Pharmacists per 100 beds (persons)	0.9183	0.1603

#### 3.2.2 Ranking results and hospital trajectories

As shown in Table 4, the composite TOPSIS scores reveal a dual pattern: overall capacity improved marginally, yet internal divergence intensified. The score range widened from [0.2667, 0.6982] in 2019 to [0.2711, 0.6199] in 2023, indicating slower progress among lagging hospitals and greater gains among leaders. Hospital A8, which fell to the bottom in 2021, surged to 1st place in 2023, implying effective strategic transformation or targeted investment in capacity domains with high weight. Hospital A10, once ranked 2nd in 2019, plummeted to last place by 2023, reflecting long-term stagnation, management inefficiencies, or weakened policy support. Hospitals A4 and A12 remained consistently above average, showing stable and balanced development across all three dimensions. These transitions suggest that secondary public hospitals can rapidly upgrade their service capacity with focused resource reallocation, though without consistent intervention, structural weaknesses persist over time.

**Table 4:** Composite service capacity scores and rankings of 12 secondary public hospitals in Guangzhou (2019–2023).

Hospital code	2019 score	2019 rank	2020 score	2020 rank	2021 score	2021 rank	2022 score	2022 rank	2023 score	2023 rank
A1	0.4348	9	0.4682	7	0.5304	6	0.5047	9	0.5486	6
A2	0.4043	10	0.4472	8	0.4424	9	0.4505	11	0.4692	9
A3	0.4776	7	0.4656	9	0.5244	7	0.5352	7	0.4961	8
A4	0.6309	2	0.6359	2	0.5943	2	0.5923	2	0.6176	2
A5	0.5229	5	0.5006	5	0.4743	8	0.5072	8	0.5046	7
A6	0.2667	12	0.3359	11	0.4012	10	0.4329	12	0.4385	11
A7	0.6982	1	0.6185	3	0.5934	3	0.5836	3	0.5982	3
A8	0.4917	6	0.4982	6	0.2932	12	0.5136	6	0.6199	1
A9	0.4138	8	0.4193	10	0.4284	11	0.5396	5	0.5569	5
A10	0.6678	3	0.6636	1	0.6081	1	0.5949	1	0.2711	12
A11	0.6065	4	0.5824	4	0.5882	4	0.5612	4	0.5828	4
A12	0.5193	6	0.5152	5	0.5406	5	0.5503	5	0.5702	5

#### 3.2.3 Kernel density distribution and polarization trends

**Figure 1:**
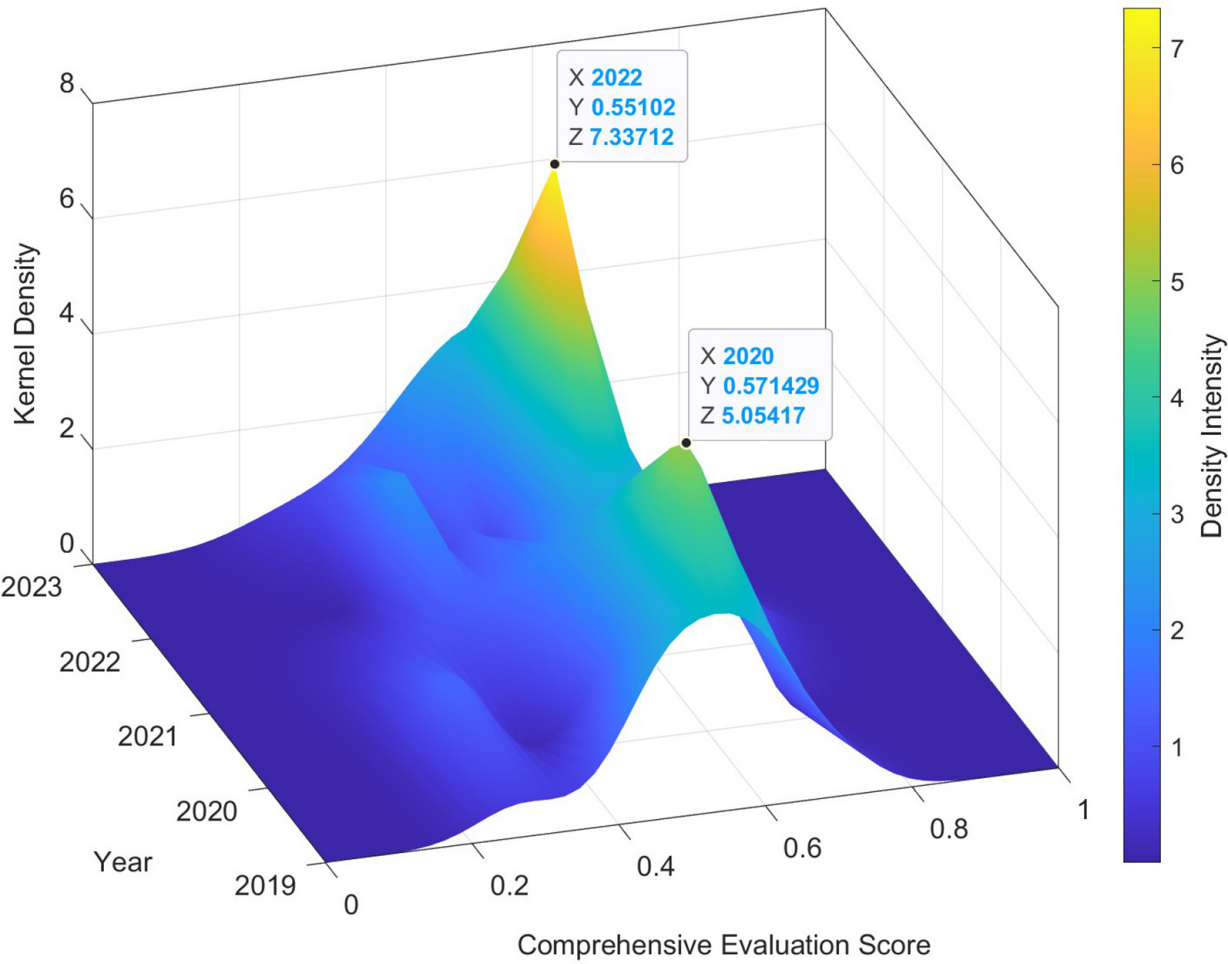
3D Kernel Density Estimation of composite service capacity scores for 12 secondary public hospitals in Guangzhou (2019–2023).

To analyze the dynamic evolution of service capacity across institutions, a 3D Kernel Density Estimation (KDE) was constructed using MATLAB, based on composite TOPSIS scores from 2019 to 2023. As shown in Figure 1, the distribution of scores has undergone significant structural changes. In 2019–2020, the KDE surface displays a unimodal and symmetric peak, centered around the 0.4–0.6 interval. This reflects a relatively cohesive system, with most hospitals clustered around a moderate level of service capacity. Starting in 2021, under the continued impact of the COVID-19 pandemic, the distribution began to diverge. A portion of hospitals improved significantly—moving above 0.6—while others fell behind. This shift produced a bimodal distribution, signaling the initial onset of performance polarization. By 2022–2023, the divide had further intensified. A new density peak formed in the 0.6–0.8 range, indicating the rise of high-performing institutions. Meanwhile, a persistent group of hospitals remained in the <0.4 score range, suggesting stagnation at the lower end. These changes point to a clear dual structure in institutional performance: top-tier hospitals advanced rapidly, while bottom-tier facilities failed to catch up. The density peaks grew sharper during 2021–2022, suggesting accelerated reordering of institutional capacity. Although the overall curve shifted rightward in 2023, indicating general improvement, the expanded variance suggests growing internal stratification across the hospital system. This pattern underscores the urgency for differentiated policy support: capacity enhancement strategies for lagging hospitals, and performance stabilization and innovation support for emerging leaders.

### 3.3 Regional disparity analysis via Dagum Gini decomposition

#### 3.3.1 Regional division principles

To systematically assess spatial differences in healthcare service capacity, the 12 secondary public hospitals in Guangzhou were grouped according to the city's official spatial development strategy into four functional regions, each aligned with distinct policy orientations and urban development priorities:

• C: Central Administrative Core— Includes Yuexiu, Liwan, and Haizhu districts. This area represents Guangzhou's traditional urban center, housing many longstanding hospitals and administrative institutions.
• E: Eastern Innovation Corridor— Includes Tianhe, Huangpu, and Zengcheng districts. This zone serves as a technology and education hub, with growing investment in research-based healthcare facilities.
• S: Southern Bay Area Gateway— Covers Panyu and Nansha districts, representing the interface between Guangzhou and the Guangdong–Hong Kong–Macao Greater Bay Area. It features newly established or upgraded hospitals under port-oriented urban expansion policies.
• N: Northern Ecological Corridor— Includes Baiyun, Huadu, and Conghua districts. This zone focuses on ecological protection and suburban development, where many hospitals serve sparsely distributed populations.

These divisions reflect Guangzhou's “multi-center, polycentric spatial layout”, which underpins its urban healthcare planning. Such classification allows for a more accurate analysis of service capacity disparities by spatial typology and functional mandate.

#### 3.3.2 Temporal patterns and inequality dynamics

**Figure 2:**
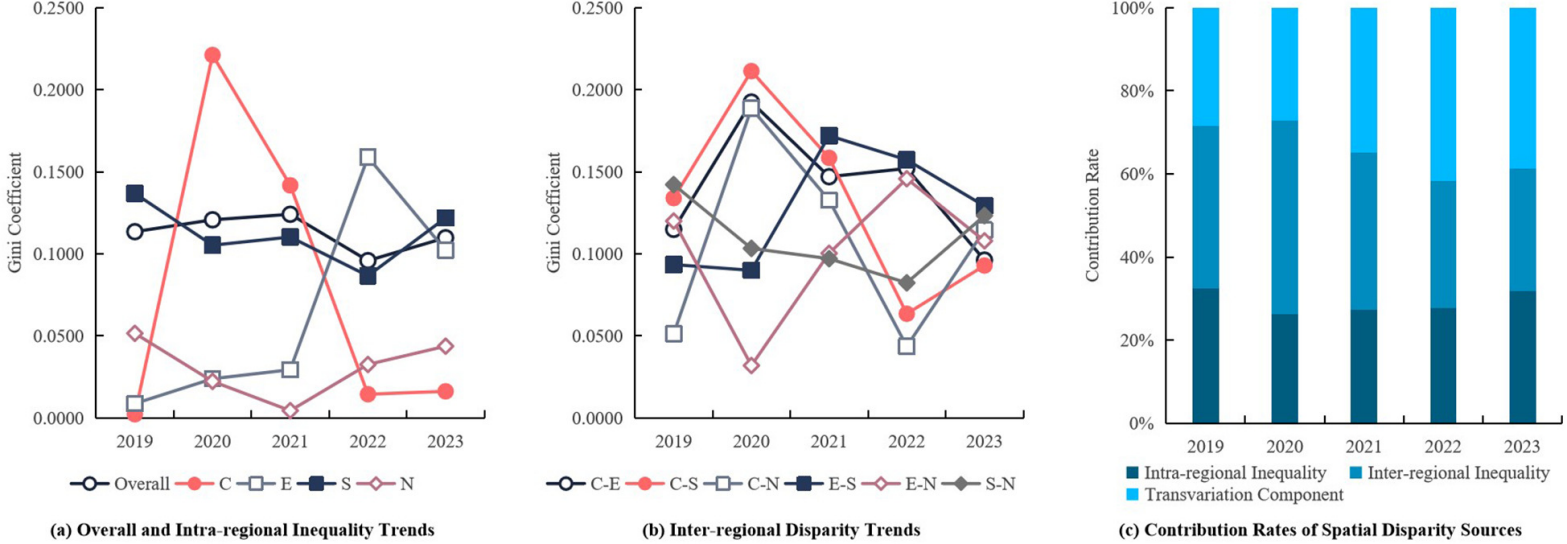
Decomposition of regional inequality in service capacity among secondary public hospitals in Guangzhou (2019–2023).

Using the Dagum Gini coefficient and its subgroup decomposition, Table 5 and Figure 2 provide insight into the spatial structure of inequality in service capacity from 2019 to 2023.The overall inequality index (G) remained relatively stable, with a notable peak in 2021 (G ≈ 0.1241), reflecting structural shocks during the mid-phase of the COVID-19 pandemic. By 2023, G slightly rebounded after a temporary decline in 2022, indicating partial re-concentration of capacity. The Southern Gateway (S) region consistently displayed the highest intra-regional inequality. This reflects the uneven development between Panyu's mature hospitals and Nansha's emerging but still under-resourced institutions. The area's transitional status—between metropolitan core and new development frontier—has led to fragmented service capability. In contrast, the Central Core (C) experienced a sharp but short-lived surge in internal disparity in 2020, likely due to extreme performance variations in a few large institutions during the pandemic. The Eastern (E) and Northern (N) zones maintained relatively balanced internal distributions, though E-region scores surged in 2022, suggesting successful infrastructure upgrades in one or more hospitals in the science-tech corridor.

**Table 5:** Decomposition of Dagum Gini coefficients and inequality components of hospital service capacity in four functional regions of Guangzhou (2019–2023).

Component	Subcomponent/Region pair	2019	2020	2021	2022	2023
Total Gini soefficient (G)	—	0.1135	0.1208	0.1241	0.0959	0.1098
Within-region inequality ($G_{w}$)	Central Administrative Core (C)	0.0023	0.2212	0.1418	0.0145	0.0162
	Eastern Innovation Corridor (E)	0.0088	0.0239	0.0294	0.159	0.1021
	Southern Bay Area Gateway (S)	0.1368	0.1053	0.1103	0.0866	0.122
	Northern Ecological Corridor (N)	0.0516	0.0223	0.0045	0.0326	0.0438
Between-region inequality ($G_{nb}$)	C–E	0.115	0.1925	0.1471	0.1521	0.0962
	C–S	0.134	0.2114	0.1585	0.0635	0.0929
	C–N	0.0513	0.1887	0.1328	0.0437	0.1145
	E–S	0.0935	0.09	0.172	0.1573	0.1292
	E–N	0.12	0.032	0.1004	0.1458	0.1079
	S–N	0.1422	0.1034	0.0971	0.0824	0.1233
Contribution rates ($G_{z}$, %)	Within-region	32.38%	26.27%	27.37%	27.70%	31.78%
	Between-region	39.19%	46.61%	37.79%	30.58%	29.45%
	Transvariation (Overlapping Density)	28.43%	27.11%	34.84%	41.73%	38.77%

#### 3.3.3 Inter-Regional disparity and component contributions

Figure 2B illustrates the inter-regional Gini coefficients between the six pairwise region combinations (e.g., C–S, C–E, E–S).The most pronounced inequality occurred in C–S and C–E during 2020, driven by differences in surge capacity and infection control preparedness between central and peripheral areas. The E–S combination maintained high inequality levels through 2021–2022, suggesting delayed convergence between the innovation belt and southern port corridor. By 2023, all inter-regional gaps showed a decline, indicating improved coordination and policy harmonization under post-pandemic reforms. Figure 2C shows the annual contributions of inequality components. Inter-regional inequality (Gnb) accounted for ∼47% in 2020, but decreased steadily to under 30% in 2023, implying that regional gaps are narrowing. Intra-regional inequality (Gw) increased slightly in 2023 (∼32%), pointing to emerging fragmentation within zones, especially in S and E regions. Most notably, the transvariation component (Gt)—reflecting overlapping capacity differences across hospitals from different zones—rose significantly after 2022, exceeding 40% in 2023, and became the dominant inequality source. This shift reveals that inequality is no longer driven solely by spatial location, but increasingly by individual institutional divergence, regardless of region. It highlights the importance of hospital-specific reforms, such as digital infrastructure, workforce incentives, and clinical specialization, over region-wide structural investment alone.

## 4 Discussion

Based on panel data from 12 secondary public hospitals in Guangzhou between 2019 and 2023, this study utilized entropy-weighted TOPSIS, Kernel Density Estimation, and Dagum Gini decomposition to comprehensively assess the temporal evolution and spatial disparities in hospital service capacity. Results indicate a general improvement in performance across medical quality, operational efficiency, and sustainability dimensions. However, these gains were accompanied by increasing internal divergence and persistent regional inequality. In light of international evidence and China's ongoing healthcare reforms, this section discusses critical challenges and future policy directions across four domains: problem identification, capacity development strategies, governance pathways, and institutional resilience.

### 4.1 Key issues and systemic challenges

#### 4.1.1 Medical quality: efficiency–safety trade-offs and functional regression

In the quality dimension, Guangzhou's secondary hospitals exhibited signs of a trade-off between treatment efficiency and patient safety. The prolonged average length of stay ($X_{1}$)—particularly in hospitals like A3 and A10—may partially reflect COVID-19 protocols but also indicates poor bed turnover and inefficient case management. The significant decline in surgical case ratios ($X_{3}$) suggests a functional hollowing effect due to tertiary hospital siphoning and insufficient technical capabilities within secondary hospitals. This nationwide trend, if unchecked, could lead to the erosion of core competencies in secondary-level care (39). While the improvement in mortality among low-risk patients ($X_{2}$) reflects better basic care quality, the fluctuating surgical complication rate ($X_{4}$) underscores ongoing weaknesses in perioperative safety and clinical governance.

#### 4.1.2 Operational efficiency: cost-structure imbalances and fiscal stress

Hospitals face mounting tension between cost containment and revenue structure optimization. For instance, A7 and A11 reported over 40% annual growth in outpatient and drug costs ($X_{8}$/$X_{9}$), highlighting a problematic reliance on pharmaceuticals and diagnostics, contrary to the policy goals of the “restructuring revenue mix” reform agenda. Although declining inpatient costs ($X_{10}$/ $X_{11}$) suggest effective insurance payment control, excessive cost-cutting may compromise diagnostic scope and financial sustainability, as seen in A3's declining rankings. Hospitals with high asset–liability ratios (e.g., A7 reaching 75%) face sustainability risks, especially when paired with declining personnel expenditure ratios ($X_{6}$), which reflect a dilemma: lower spending does not necessarily yield higher efficiency.

#### 4.1.3 Sustainability: workforce structure and professional burnout

In terms of long-term sustainability, persistent workforce structural weaknesses hinder the development of resilient service capacity. While the nurse-to-doctor ratio ($X_{12}$) has improved overall (reaching 0.734), understaffed pharmacy units ($X_{14}$) remain a barrier to pharmaceutical services and rational drug use. Unequal physician inpatient workloads ($X_{13}$)—exceeding two beds per day in hospitals like A6 and A10—may lead to burnout and undermine care consistency. These personnel imbalances reveal fundamental constraints in human resource planning that could hinder hospitals' adaptive capacity in the face of public health crises.

#### 4.1.4 Spatial disparities: fragmentation and cross-regional competition

The spatial pattern of healthcare service capacity has become increasingly fragmented. The Southern Bay Area Gateway (S) exhibited the highest intra-regional inequality (Gw = 0.1220), driven by gaps between newly established hospitals in Nansha and mature institutions in Panyu. Moreover, the rise in transvariation contribution (Gz = 38.77%) indicates intensified cross-regional overlap in hospital performance. For example, Hospital A8 (Eastern Innovation Corridor) and Hospital A4 (Central Core) now compete in overlapping patient markets, reflecting unbalanced spatial allocation of resources and system-wide coordination challenges. These trends call for a dual focus on regional integration and differentiated policy design, to both strengthen underperforming institutions and foster synergy across urban functional zones. A more equitable and resilient health system requires nuanced governance beyond mere expansion of infrastructure.

### 4.2 Strategic pathways for capacity optimization

Drawing on the findings of this study, a coherent reform strategy is essential to enhance the capacity, resilience, and coordination of secondary public hospitals in Guangzhou. Key insights suggest that while service quality has generally improved, operational efficiency remains uneven, and structural challenges persist in sustainability.

#### 4.2.1 Quality-led differentiated development

The evaluation results show diverging trends in quality indicators: while low-risk mortality rates declined and complication rates slightly improved, average length of stay increased, and surgical discharge rates declined, especially in hospitals like A3 and A10. These trends suggest a weakening of procedural functionality and the fragmentation of surgical capacity in some institutions—possibly due to case siphoning by tertiary centers or internal capability stagnation. To address these issues, reforms should: Designate clinical specialization tracks for underperforming hospitals—e.g., those with high length of stay, low surgical output, or redundant catchment areas—to evolve into focused hubs in areas like rehabilitation or geriatrics; Define eligibility thresholds using capacity metrics (e.g., surgical discharge rate <20%, stay >9 days, or domain-specific staffing strengths); Align with national zoning and global best practices, such as China's Health Service Plan, NHS trust models, and Mayo's hub-and-spoke frameworks, to enhance service clarity and resource efficiency.

#### 4.2.2 Efficiency-oriented financial and human resource governance

The divergence in outpatient and drug cost growth in 2023 (over 40% in some hospitals) reflects a growing gap in cost control capabilities. Asset-liability ratios also indicate uneven financial health, with some hospitals approaching high-risk thresholds (40). To address this, reforms should: Standardize cost governance mechanisms, including bundled payments and global budgeting pilots; Adjust personnel expenditure structures, rewarding performance while ensuring workforce retention; Build digital financial dashboards to allow real-time monitoring of cost drivers, flagging anomalies in outpatient and pharmaceutical expenses.

#### 4.2.3 Sustainability-focused workforce and institutional resilience

Human resource data reveal a stable yet suboptimal trajectory: the nurse-to-doctor ratio has slightly improved (from 0.713 to 0.734), and physician workload shows minor relief. However, pharmacist staffing has declined, raising concerns about pharmaceutical governance. Therefore: Revise workforce standards to reflect differentiated service needs across specialties; Incentivize pharmacy and public health professionals to counterbalance current personnel imbalances; Embed resilience benchmarks into hospital evaluations—e.g., surge capacity, staffing flexibility, and supply chain autonomy.

These pathways align with the broader goals of China's hierarchical medical system reform and the WHO's call for resilient, integrated health systems. Through differentiated development, refined financial governance, and targeted workforce strategies, secondary hospitals can better fulfill their role as stable, adaptable, and equitable pillars within urban health systems.

### 4.3 Implementation roadmap

**Figure 3:**
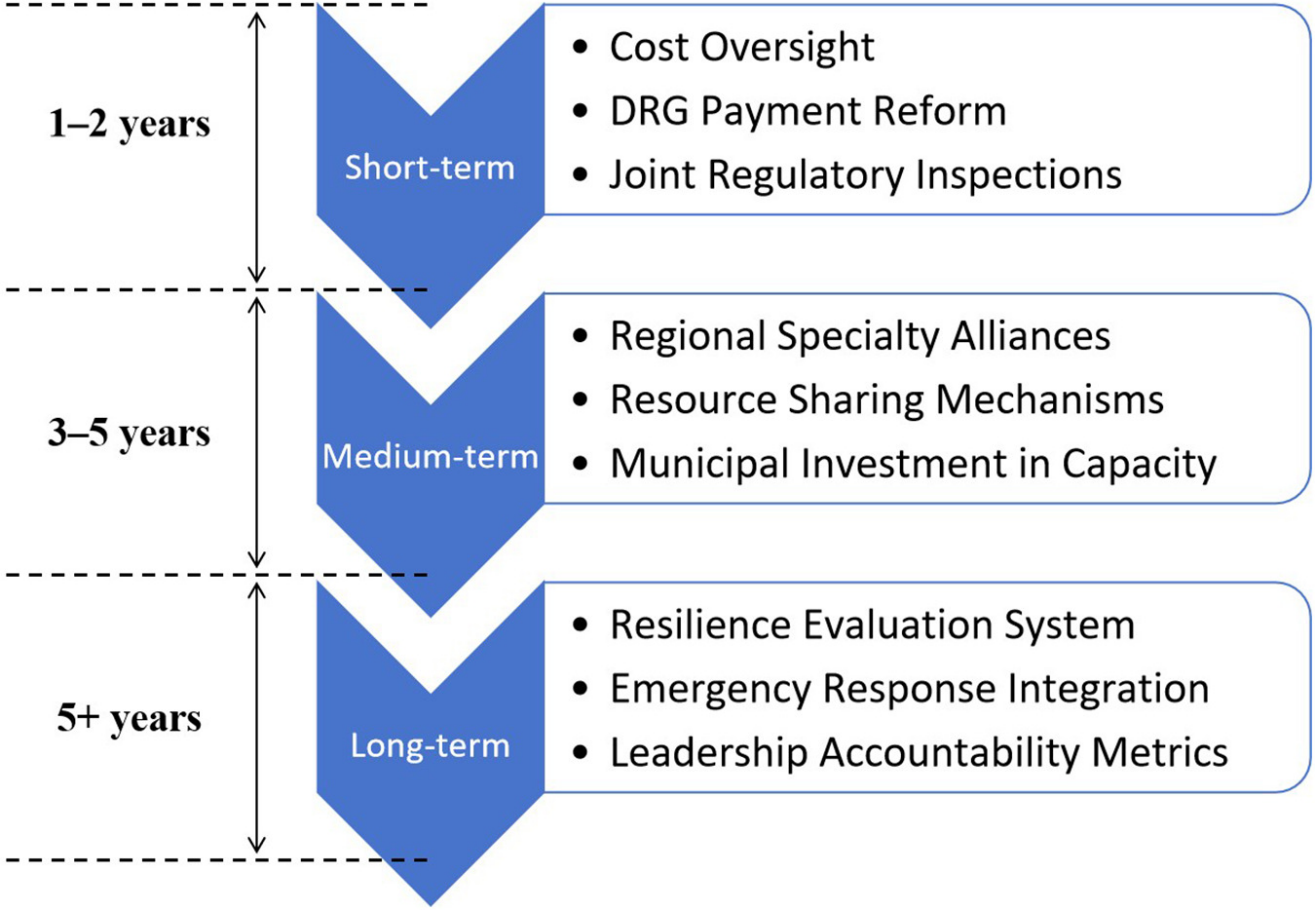
Strategic roadmap for implementing capacity enhancement in secondary public hospitals.

To ensure policy effectiveness, the proposed capacity enhancement strategies should be implemented in a phased manner—short-term, medium-term, and long-term—tailored to institutional readiness and policy capacity. Figure 3 presents a strategic roadmap that outlines the temporal and functional dimensions of reform.

Short-term (1–2 years): Target hospitals with abnormal cost growth for focused remediation. Leverage DRG-based payment reform as an entry point. Joint inspections by the Health Commission and Medical Insurance Bureau should be conducted, with penalties such as budget reductions for non-compliant institutions. These actions will help restore expenditure discipline and enhance accountability.

Medium-term (3–5 years): Establish 3–5 regional specialty alliances to optimize resource allocation and improve professional collaboration. Municipal funding should support equipment upgrades and pharmacist deployment across alliance hospitals. Resource sharing will enhance service integration across zones.

Long-term (5+ years): Develop a resilience evaluation framework for hospitals that includes emergency response capacity. Performance in crisis response and service conversion should be incorporated into hospital leadership assessments, enabling hospitals to evolve in both “routine” and “emergency” dimensions. This dual-track capacity model ensures system-wide adaptability and public health readiness (41).

### 4.4 Potential challenges and strategic countermeasures

While well-designed, the implementation of these strategies may encounter multiple barriers, including fiscal constraints, data silos, and institutional inertia. Given limited public finance, local governments could issue dedicated municipal health bonds, prioritizing investment in high-weight indicators such as surgical capability and pharmacist allocation. This ensures targeted reinforcement of structural bottlenecks. Fragmented information systems across the Medical Insurance Bureau, Health Commission, and Human Resources departments hinder policy synergy. Authorities should develop a real-time data visualization platform based on KDE, enabling dynamic surveillance, early warnings, and resource redeployment. Resistance to role reform among medical staff remains a persistent problem. Reforming promotion and evaluation systems to include indicators such as surgical volume, grassroots service, and technical outreach will help incentivize downward mobility and skill transfer to community settings, thereby improving service equity and accessibility (39).

## 5 Conclusion

This study investigates the evolution of service capacity across 12 secondary public general hospitals in Guangzhou from 2019 to 2023. A multidimensional evaluation framework was constructed, incorporating four core dimensions: medical quality, operational efficiency, sustainability, and regional coordination. By integrating the entropy-weighted TOPSIS method, Kernel Density Estimation (KDE), and Dagum Gini decomposition, the analysis revealed the spatiotemporal dynamics of institutional performance and inequality.

The results suggest that while overall service capacity has improved, significant inter-hospital and inter-regional disparities persist. Key structural bottlenecks—particularly in surgical capability and pharmacist staffing—remain critical obstacles to realizing the functional positioning and high-quality development of secondary hospitals.

At the methodological level, this research contributes an innovative modeling approach, validating the combined application of entropy-TOPSIS and Dagum Gini decomposition in capturing spatial divergence and performance trends. The proposed strategy emphasizes a dual-pathway approach: precision-targeted capacity enhancement, and adaptive governance, incorporating DRG-based payment reform, KDE-driven real-time monitoring, regional specialty alliance building, and human resource restructuring. These policy recommendations aim to strike a balance between efficiency, equity, and resilience.

In terms of global relevance, the study outlines a potential “Guangzhou Model” that could inform capacity-building in urbanizing middle-tier hospital systems across Southeast Asia and other fast-growing urban regions.

Nonetheless, several limitations remain. First, the current indicator system is primarily structural and does not capture subjective measures such as patient satisfaction or staff experience. Future research should incorporate survey-based metrics to improve sensitivity and completeness. Second, methodological expansion is needed—e.g., introducing machine learning techniques like random forests for predictive modeling and scenario classification. Third, the strategic pathways proposed in this study have not undergone longitudinal validation. Future evaluations should focus on dynamic monitoring of reform implementation, particularly the long-term effects of regional specialty alliances on surgical volume, cost structure, and service quality post-2025.

In conclusion, strengthening the capacity of secondary public hospitals in Guangzhou is not only vital for internal optimization of the healthcare system but also foundational to the efficiency of hierarchical diagnosis and treatment and the realization of equitable, accessible healthcare. Moving forward, a data-driven, problem-oriented, and regionally coordinated strategy is essential to support the long-term development of a modernized Chinese healthcare system.

## Data Availability

The original contributions presented in the study are included in the article/Supplementary Material, further inquiries can be directed to the corresponding author.
